# Primary tumor resection improves survival in patients with multifocal intrahepatic cholangiocarcinoma based on a population study

**DOI:** 10.1038/s41598-021-91823-x

**Published:** 2021-06-09

**Authors:** Linlin Yin, Si Zhao, Hanlong Zhu, Guozhong Ji, Xiuhua Zhang

**Affiliations:** Medical Center for Digestive Diseases, Second Affiliated Hospital, Nanjing Medical University, Nanjing, China

**Keywords:** Hepatology, Liver, Surgical oncology, Cancer therapy

## Abstract

The purpose of our study was to evaluate the effect of surgery on the survival and prognosis of patients with multifocal intrahepatic cholangiocarcinoma (ICCA). Patients with multifocal ICCA were selected from the SEER (Surveillance, Epidemiology, and End Results) database between 2010 and 2016. Kaplan–Meier analyses and log-rank tests were used to evaluate the difference in survival between the surgery group and the non-surgery group. We applied the Cox proportional hazards regression model to identify prognostic factors of overall survival (OS) and cancer-specific survival (CSS). In total, 580 patients were enrolled in our study, including 151 patients who underwent surgery and 429 patients who did not. The median survival time of surgical patients was longer than non-surgical patients (OS: 25 months vs. 8 months, p < 0.001; CSS: 40 months vs. 25 months, p < 0.001). Similarly, the 5-year survival rate in the surgery group was significantly higher than those in the non-surgery group (5-year OS rate: 12.91% vs. 0%; p < 0.001; 5-year CSS rate:26.91% vs. 0%; p < 0.001). Multivariate Cox analysis showed that the OS (HR:0.299, 95% CI: 0.229–0.390, p < 0.001) and CSS (HR:0.305, 95% CI:0.222–0.419, p < 0.001) of patients undergoing surgical resection were significantly improved. Meanwhile, after propensity score matching (PSM) of the original data, we come to the same conclusion.

## Introduction

Cholangiocarcinoma (CCA) is a heterogeneous biliary carcinoma (BTC) originating from intrahepatic and extrahepatic bile duct epithelial cells^1^. According to the tumor location, CCA can be divided into the distal bile duct, perihilar bile duct, or intrahepatic bile duct^2^. Intrahepatic cholangiocarcinoma (ICCA) occurs distal to the root of the secondary bile duct, accounting for about 20% of all CCA. Compared with other BTC, ICCA has different molecular, anatomical, genomic, clinical, and prognostic characteristics^3^. Although ICCA accounts for only 3% of gastrointestinal tumors and is considered a relatively rare tumor; its incidence has increased in the western world over the past few decades^4,5^. At present, ICCA has become the second-largest primary liver cancer following hepatocellular carcinoma (HCC), comprising approximately 10%-15% of primary liver malignant tumors^6^. In the United States, the incidence of ICCA is lower than that in eastern countries, with an incidence of 1.67/100,000; however, it is predicted that primary liver cancer will become the second leading cause of death in the United States by 2030^7^.

The best treatment for ICCA is surgical resection. Unfortunately, intrahepatic cholangiocarcinoma usually has no obvious symptoms and signs (such as jaundice); therefore, patients with ICCA may develop locally advanced or metastatic diseases, missing the opportunity for surgical resection^8^. Considering the lack of effective screening strategies and the highly invasive nature of this disease, about 65% of patients with ICCA were unresectable^9,10^. Among the remaining patients who met the conditions for surgical resection, even if they successfully underwent R0 resection, the survival rate was still very low. It was reported that the 5-year survival rate was 30–40%, while the 5-year survival rate of patients with lymph node metastasis was even more frustrating at about 20%^5,11^.

Previous studies have shown that up to 48% of patients with intrahepatic cholangiocarcinoma may develop multiple intrahepatic lesions before distant metastasis^12^. Multifocal ICCA was classified as T2b in the T staging of AJCC v.7 (the American Joint Committee on Cancer, the 7th Edition); in the TNM staging, the T2b category without lymph node metastasis was classified as Stage II disease or assigned to Stage IVa disease (if N1) (Supplementary Table S1). In the study of Lamarca et al., patients with multiple lesions in the liver had a worse prognosis, regardless of N status; they suggested that multifocal ICCA should be assigned to Stage IVa^12,13^. Because multiple lesions usually reflect the hematogenous intrahepatic spread (liver metastasis) from a primary predominant tumoral liver lesion, and the clinical prognosis is expected to be poor, more similar to M1 disease than to early stages. Also, in the new AJCC.v8 staging, which was published in 2016 and made effective in 2018, experts ignored the poor prognosis of ICCA with multiple lesions. In AJCC.v8, multifocal ICCA without lymphatic metastasis was classified as Stage II, while patients (if N1) were divided into Stage IIIb (Supplementary Table S1).

For patients with multifocal ICCA, palliative treatment rather than surgery is usually used^14^. Therefore, it is not clear whether surgical intervention can also improve the survival and prognosis of such patients. Due to the relatively low incidence of intrahepatic cholangiocarcinoma, we chose the SEER database, which may provide us with more detailed and accurate results for research and analysis.

## Methods

### Database and patient selection

We conducted a retrospective survey of patients with primary intrahepatic cholangiocarcinoma in the SEER database from 2010 to 2016. The SEER database contains information about patient demographics, cancer incidence and prevalence, tumor characteristics, treatment and mortality; its data come from 19 United States regions, accounting for about 34 percent of the total population. Detailed information about the tumor can be obtained from the relevant software (SEER ∗ Stat software, Version 8.3.8) provided by SEER. The patients with multifocal intrahepatic cholangiocarcinoma included in our study were classified according to AJCC.v7 staging. We selected all patients with the third Edition of the International Classification of Diseases for Oncology (ICD-O-3) site code 8160 /3. We set the following exclusion criteria: (1) Non-T2b patients and lack of TNM staging information; (2) patients with distant metastasis; (3) patients who lacked surgery-related information; (4) patients whose survival time was 0 months or unknown.

### Covariates and outcomes

The data extracted from the database include demographics (age, race, gender, marital status, and insurance status at the time of diagnosis), cancer characteristics (tumor size, lymph node status, tumor grade, vascular invasion, multiple liver lobes, survival time, vital status, and cause of death) and treatment (primary tumor resection, chemotherapy, and radiotherapy). According to the primary tumor resection, the patients were subdivided into the operation group and the non-surgery group. At the same time, the age variable was divided into < 65 or ≥ 65 years old, and the tumor size variable was divided into four groups: < 5 cm, 5–10 cm, > 10 cm, and unknown size.

The primary outcome of our study was the overall survival (OS) and cancer-specific survival (CSS). In survival analysis, the overall survival (OS) was calculated according to the interval from diagnosis to death or the last screening; CSS was defined as the interval from diagnosis to death attributed to multifocal intrahepatic cholangiocarcinoma or the last follow-up.

### Statistical analysis

Among the variables we included, classified variables were expressed as percentages, and continuous variables were expressed as medians; chi-square test (or Fisher's exact test) and student’s t-test were used to compare the differences in demography and tumor biological characteristics between the two groups. We drew the Kaplan–Meier survival curve and used the log-rank test to compare the survival difference between the operation group and the non-operation group. In addition, we also carried out subgroup analysis according to the variables we included (age, race, gender, marital status, insurance status, tumor size, lymph node status, tumor grade, vascular invasion, multiple liver lobes, radiotherapy, and chemotherapy) to evaluate which patients were more likely to benefit from the surgery. The median survival time, 1-, 3- and 5-year survival rate of each group were calculated by the Kaplan–Meier method. Meanwhile, for the regression survival analysis of patients with multifocal ICCA, we preliminarily used univariate Cox regression analysis to screen variables, excluding the variables with p > 0.05 and incorporating the remaining variables into the multivariate cox regression model. Finally, we used propensity score matching (PSM) to overcome the covariates' different distributions between the two groups and then further compared the survival difference. Student’s t-test, chi-square test, and cox regression analysis were carried out using SPSS22 (IBM Corp, Armonk, NY) statistical software. GraphPad Prism 9.0 (GraphPad Software, San Diego, CA) was used for the Kaplan–Meier survival curve and the log-rank test. PSM was performed using R 4.0.2(Institute for Statistics and Mathematics, Vienna, Austria).

### Ethics statement

Our data came from the SEER database and signed a data agreement (15718-Nov2019), so our study was exempt from ethical review. This article does not contain any studies with human participants performed by any of the authors.

## Results

### Demographic and tumor characteristics

**Figure 1:**
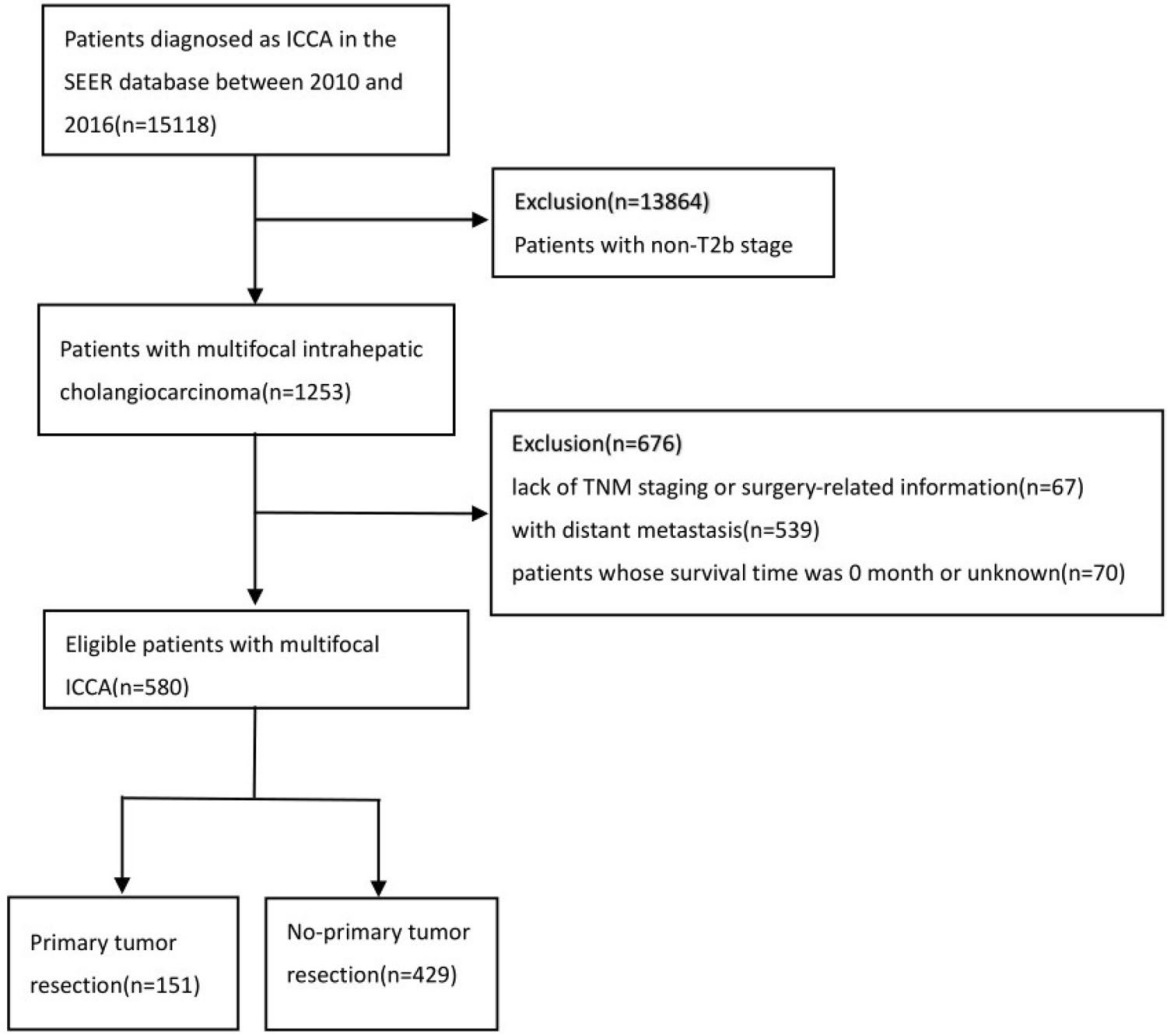
Flow diagram of eligible patients diagnosed with multifocal intrahepatic cholangiocarcinoma.

After several rounds of screening, 580 patients were enrolled in this study, including 151 patients who underwent surgery and 429 patients who did not. The detailed process is shown in Fig. 1. There were significant differences in most baseline characteristics between the surgery and non-surgery groups, such as insurance status, tumor grade, vascular invasion, multiple hepatic lobe involvement, and tumor size (Table 1). Compared with patients who did not perform primary tumor resection, patients in the resection group tended to have insurance (93.4% vs.83.0%, p = 0.007), had a higher proportion of tumor Grade I-II (47.0% vs. 18.4%, p < 0.001), vascular invasion (37.1% vs. 23.1%, p = 0.001), single liver lobe (74.2% vs. 41.5%, p < 0.001) and tumor size < 5 cm (38.4% vs. 16.3%, p < 0.001).

**Table 1:** Baseline characteristics of patients with multifocal intrahepatic cholangiocarcinoma.

Variables	Total n = 580	Primary tumor resection n = 151	No primary tumor resection n = 429	p-value
**Age group, years, %**				
< 65	277 (47.7%)	78 (51.6%)	199 (46.4%)	0.265
≥ 65	303 (52.3%)	73 (48.4)	230 (53.6%)	
**Race, %**				
White	449 (77.4%)	117 (77.5%)	332 (77.4%)	0.717
Black	54 (9.3%)	12 (7.9%)	42 (9.8%)	
Others	77 (13.3%)	22 (14.6%)	55 (12.8%)	
**Gender, %**				
Male	264 (45.6%)	69 (45.6%)	195 (45.5%)	0.959
Female	316 (54.4%)	82 (54.4%)	234 (54.5%)	
**Insurance status, %**				
Uninsured	76 (13.1%)	9 (6.0%)	67 (15.6%)	0.007
Insured	497 (85.7%)	141 (93.4%)	356 (83.0%)	
Unknown	7 (1.2%)	1 (0.6%)	6 (1.4%)	
**Marital status, %**				
Unmarried	209 (36%)	45 (29.8%)	164 (38.2%)	0.177
Married	349 (60.2%)	100 (66.2%)	249 (58.0%)	
Unknown	22 (3.8%)	6 (4.0%)	16 (3.7%)	
**Grade, %**				
Grade I–II	150 (25.9%)	71 (47.0%)	79 (18.4%)	< 0.001
Grade III–IV	135 (23.3%)	46 (30.5%)	89 (20.8%)	
Unknown	295 (50.9%)	34 (22.5%)	261 (60.8%)	
**LN metastases, %**				
No	423 (72.9%)	117 (77.5%)	306 (71.3%)	0.143
Yes	157 (27.1%)	34 (22.5%)	123 (28.7%)	
**Chemotherapy, %**				
No	234 (40.3%)	61 (40.4%)	173 (40.3%)	0.988
Yes	346 (59.7%)	90 (59.6%)	256 (59.7%)	
**Radiation, %**				
No	492 (84.8%)	131 (86.8%)	361 (84.1%)	0.443
Yes	88 (15.2%)	20 (13.2%)	68 (15.9%)	
**Vascular invasion, %**				
No	425 (73.3%)	95 (62.9%)	330 (76.9%)	0.001
Yes	155 (26.7%)	56 (37.1%)	99 (23.1%)	
**Multiple lobes, %**				
No	290 (50.0%)	112 (74.2%)	178 (41.5%)	< 0.001
Yes	290 (50.4%)	39 (25.8%)	251 (58.5%)	
**Primary tumor size, %**				
≤ 5 cm	128 (22.1%)	58 (38.4%)	70 (16.3%)	< 0.001
5–10 cm	210 (36.2%)	59 (39.1%)	151 (35.2%)	
≥ 10 cm	111 (19.1%)	27 (17.9%)	84 (19.6%)	
Unknown	131 (22.6%)	7 (4.6%)	124 (28.9%)	

To further analyze the selection tendency of surgical patients, we conducted the binary logistic regression analysis. The regression analysis results indicated that younger patients with tumor Grade I–II, tumor size < 5 cm, single liver lobe, and insurance were much more likely to perform surgery than others (Table2).

**Table 2:** Logistic regression model for receiving surgery.

Characteristic	Adjusted OR (95% CI)	p-value
**Age group, years**		
< 65	Reference	0.018
≥ 65	0.575 (0.363–0.910)	
**Race**		
White	Reference	
Black	0.924 (0.407–2.101)	0.851
Others	1.099 (0.567–2.129)	0.779
**Gender**		
Female	Reference	0.945
Male	0.984 (0.623–1.554)	
**Insurance status**		
Uninsured	Reference	
Insured	3.312 (1.455–7.536)	0.004
Unknown	2.412 (0.187–31.062)	0.5
**Marital status**		
Unmarried	Reference	
Married	1.283 (0.786–2.095)	0.319
Unknown	0.948 (0.271–3.316)	0.934
**Grade**		
Grade I–II	Reference	
Grade III–IV	0.173 (0.102–0.294)	< 0.001
Unknown	0.632 (0.369–1.085)	< 0.096
**LN metastases**		
No	Reference	0.759
Yes	0.921 (0.554–1.558)	
**Vascular invasion**		
No	Reference	0.062
Yes	1.568 (0.978–2.512)	
**Multiple lobes**		
No	Reference	< 0.001
Yes	0.286 (0.180–0.455)	
**Primary tumor size**		
≤ 5 cm	Reference	
5–10 cm	0.490 (0.287–0.837)	0.009
≥ 10 cm	0.394 (0.205–0.757)	0.005
Unknown	0.107 (0.043–0.265)	< 0.001
**Chemotherapy**		
No	Reference	0.978
Yes	0.993 (0.605–1.630)	
**Radiation**		
No	Reference	0.61
Yes	0.847 (0.447–1.604)	

### Influence of primary tumor resection on OS and CSS

**Figure 2:**
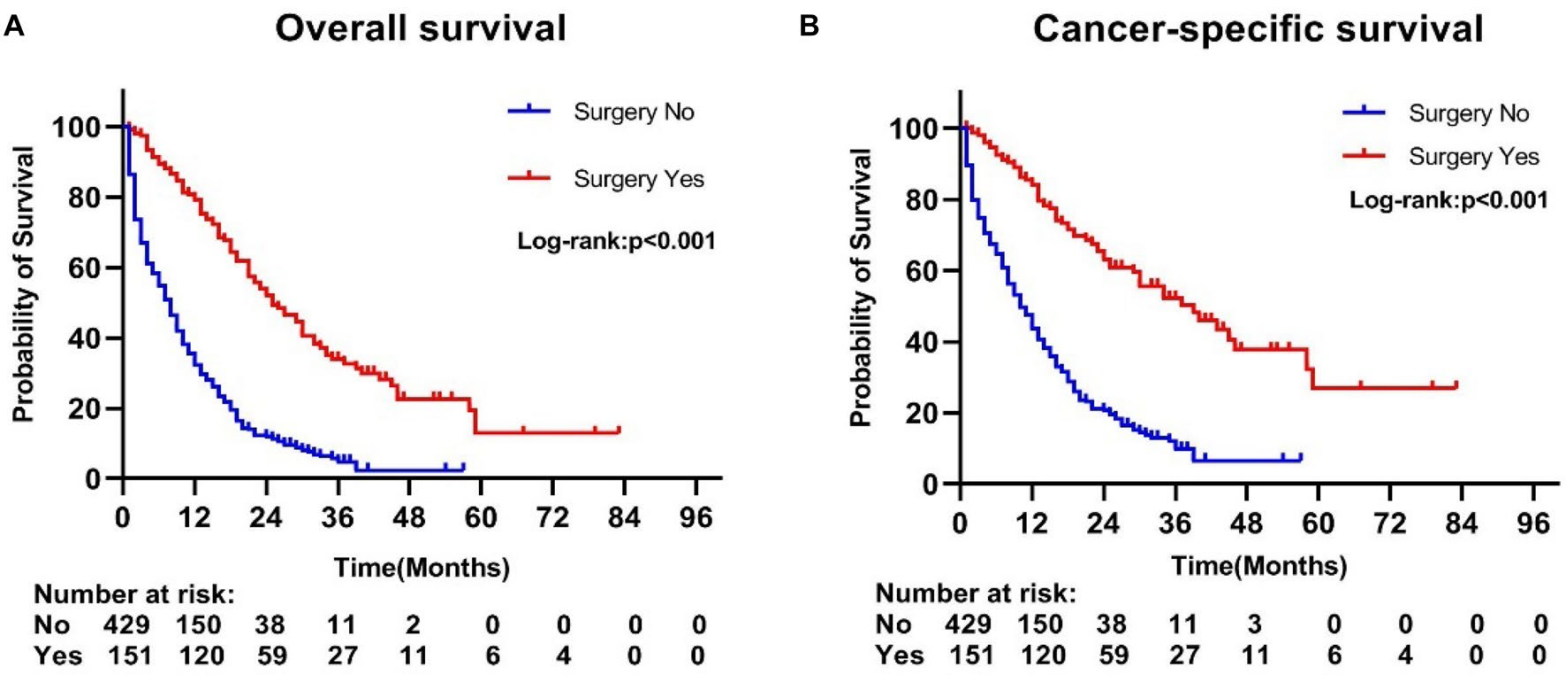
Kaplan–Meier curves of (**A**) overall and (**B**) cancer-specific survival according to whether patients underwent primary tumor surgery in the overall cohort.

Primary tumor resection significantly improved OS in multifocal intrahepatic cholangiocarcinoma patients, with a median survival of 25 months in the surgery group versus 8 months in the non-surgery group (p < 0.001). Meanwhile, there was a trend toward a higher 1-, 3- and 5-year survival rate in the surgery group compared to patients without primary tumors removed (1-year OS rate: 79.34% vs. 32.17%, 3-year OS rate: 34.01% vs. 4.72%, 5-year OS rate: 12.91% vs. 0%; p < 0.001). The comparison of survival curves between the two groups is shown in Fig. 2A. Likewise, we have concluded a similar conclusion in the comparison of CSS between the surgical and non-surgical groups. Patients with surgery were associated with a significantly high likelihood of longer median survival time than the non-resection group (39 months vs. 10 months, p < 0.001). In addition, there was a significant difference in the 1-, 3- and 5-year cancer specific-survival rate between the operation group and the non-operation group (1-year CSS rate: 84.14 vs. 43.66%, 3-year CCS rate: 52.33% vs. 9.83%, 5-year CSS rate: 26.91% vs. 0%; p < 0.001), CSS curves as shown in Fig. 2B.

### Subgroup analysis in the surgery group

We performed a subgroup analysis in the resection group to further evaluate which patients were more likely to benefit from the surgery. Patients were divided into multiple subgroups stratified by age, race, gender, marital status, insurance status, tumor size, lymph node status, tumor grade, vascular invasion, multiple liver lobes, radiotherapy, and chemotherapy. The results revealed that the OS of Age < 65 years old was improved compared to Age ≥ 65 years old (median survival time: 30 months vs. 22 months, p = 0.0195); the OS of women was significantly longer than men (median survival time: 30 vs. 19 months, p = 0.0027); the prognosis of patients with tumor Grade I–II was also better than grade III–IV (median survival time: 30 months vs. 22 months, p = 0.029); and patients without lymph node metastasis were more likely to benefit from surgery (median survival time: 30 vs. 18 months, p = 0.0068) (Supplementary Table S2). For the CSS of each subgroup, we found that black people, males, patients with lymph node metastasis or tumor size > 10 cm had a worse survival prognosis after primary tumor resection (Supplementary Table S3).

### Predictors of survival

To find out the independent prognosis and risk factors of OS or CSS, we performed Cox regression analysis. We first screened the variables with p < 0.05 in univariate Cox regression analysis and then included the selected variables into the multivariate Cox regression model. The univariate Cox regression results of OS are shown in Table 3. We excluded three variables: marital status, vascular invasion, and lymph node metastasis (p > 0.05). In our multivariate analysis, we found that primary tumor resection (HR: 0.299, 95% CI: 0.229–0.390, p < 0.001), chemotherapy (HR: 0.547, 95% CI: 0.451–0.664, p < 0.001) and radiotherapy (HR: 0.542, 95% CI: 0.415–0.708, p < 0.001) were beneficial to reduce the overall risk of death, while black population (HR: 1.458, 95% CI: 1.078–1.973, p = 0.015), male (HR: 1.265, 95% CI: 1.051–1.522, p = 0.013) and tumor Grade III-IV (HR: 1.553, 95% CI: 1.178–2.047, p = 0.002) were independent risk factors decreasing OS (Table 3). When analyzing cancer-specific survival, 7 variables (race, gender, tumor size, tumor grade, multiple liver lobes, radiotherapy, and chemotherapy) were included in the multivariate Cox regression model; five variables (age, marital status, insurance status, lymph node status and vascular invasion) were excluded (Table 4). In multivariate Cox regression model, primary tumor resection (HR: 0.305, 95%CI: 0.222–0.419, p < 0.001), chemotherapy (HR: 0.607, 95% CI: 0.483–0.762, p < 0.001) and radiotherapy (HR: 0.556, 95% CI: 0.409–0.756, p < 0.001) were still independent prognostic factors to improve cancer-specific survival, and black race (HR:1.564, 95% CI:1.103–2.218, p = 0.012), male (HR:1.315, 95% CI:1.060–1.631, p = 0.012), tumor Grade III-IV (HR: 1.910, 95% CI: 1.364–2.675, p < 0.001), tumor size > 10 cm (HR: 1.433, 95% CI: 1.006–2.042, p = 0.046) had poor CSS (Table4).

**Table 3:** Factors associated with overall survival of patients with multifocal intrahepatic cholangiocarcinoma.

Characteristic	Deaths, n (%)	Univariable		Multivariable	
		HR (95% CI)	p-value	HR (95% CI)	p-value
**Age group, years**					
< 65	225 (81.2)	Reference		Reference	
≥ 65	255 (84.1)	1.311 (1.095–1.571)	0.003	1.157 (0.947–1.414)	0.251
**Race**					
White	367 (81.7)	Reference		Reference	
Black	49 (90.7)	1.512 (1.121–2.039)	0.007	1.458 (1.078–1.973)	0.015
Others	64 (83.1)	1.034 (0.792–1.348)	0.807	1.073 (0.821–1.403)	0.606
**Gender**					
Female	228 (86.3)	Reference		Reference	
Male	252 (79.7)	1.353 (1.353–1.619)	0.01	1.265 (1.051–1.522)	0.013
**Insurance status**					
Uninsured	67 (88.1)	Reference		Reference	
Insured	407 (81.8)	0.683 (0.527–0.885)	0.004	0.862 (0.648–1.147)	0.396
Unknown	6 (85.7)	0.845 (0.366–1.951)	0.694	0.958 (0.402–2.279)	0.973
**Marital status**					
Unmarried	177 (84.6)	Reference			
Married	284 (81.3)	0.873 (0.723–1.053)	0.155		
Unknown	19 (86.3)	0.823 (0.512–1.321)	0.419		
**Grade**					
Grade I -II	104 (69.3)	Reference		Reference	
Grade III-IV	109 (80.7)	1.596 (1.218–2.091)	0.001	1.553 (1.178–2.047)	0.002
Unknown	267 (90.5)	2.098 (1.668–2.638)	< 0.001	1.408 (1.102–1.800)	0.006
**LN metastases**					
No	348 (82.2)	Reference			
Yes	132 (84.0)	1.108 (0.906–1.354)	0.319		
**Vascular invasion**					
No	348 (81.8)	Reference			
Yes	132 (85.1)	0.998 (0.817–1.220)	0.985		
**Multiple lobes**					
No	228 (78.6)	Reference		Reference	
Yes	252 (86.8)	1.359 (1.135–1.627)	0.001	1.169 (0.960–1.422)	0.247
**Primary tumor size**					
≤ 5 cm	92 (71.8)	Reference		Reference	
5–10 cm	172 (81.9)	1.289 (1.000–1.662)	0.05	1.231 (0.944–1.606)	0.125
≥ 10 cm	92 (82.8)	1.208 (0.904–1.613)	0.201	1.126 (0.833–1.521)	0.441
Unknown	124 (94.6)	2.089 (1.591–2.744)	< 0.001	1.510 (1.130–2.018)	0.005
**Chemotherapy**					
No	197 (84.1)	Reference		Reference	
Yes	283 (81.7)	0.667 (0.556–0.802)	< 0.001	0.547 (0.451–0.664)	< 0.001
**Radiation**					
No	413 (83.9)	Reference		Reference	
Yes	67 (76.1)	0.637 (0.492–0.825)	< 0.001	0.542 (0.415–0.708)	< 0.001
**Resection of the primary tumor**					
No	387 (90.2)	Reference		Reference	
Yes	93 (61.5)	0.306 (0.241–0.388)	< 0.001	0.299 (0.229–0.390)	< 0.001

**Table 4:** Factors associated with cancer-specific survival of patients with multifocal intrahepatic cholangiocarcinoma.

Characteristic	Deaths, n (%)	Univariable		Multivariable	
		HR (95% CI)	p-value	HR (95% CI)	p-value
**Age group, years**					
< 65	190 (68.5)	Reference			
≥ 65	165 (54.4)	0.998 (0.809–1.231)	0.985		
**Race**					
White	265 (59.0)	Reference		Reference	
Black	37 (68.5)	1.578 (1.118–2.229)	0.01	1.564 (1.103–2.218)	0.012
Others	53 (68.8)	1.185 (0.882–1.592)	0.26	1.245 (0.924–1.678)	0.15
**Gender**					
Female	184 (58.2)	Reference		Reference	
Male	171 (64.7)	1.385 (1.124–1.707)	0.002	1.315 (1.060–1.631)	0.012
**Insurance status**					
Uninsured	46 (60.5)	Reference			
Insured	305 (61.3)	0.748 (0.548–1.022)	0.068		
Unknown	4 (57.1)	0.831 (0.299–2.311)	0.723		
**Marital status**					
Unmarried	135 (64.5)	Reference			
Married	207 (59.3)	0.835 (0.672–1.038)	0.105		
Unknown	13 (59.0)	0.736 (0.416–1.302)	0.292		
**Grade**					
Grade I–II	63 (42.0)	Reference		Reference	
Grade III–IV	82 (60.7)	1.984 (1.426–2.760)	< 0.001	1.910 (1.364–2.675)	< 0.001
Unknown	210 (71.1)	2.728 (2.052–3.626)	< 0.001	1.839 (1.359–2.488)	< 0.001
**LN metastases**					
No	250 (59.1)	Reference			
Yes	105 (66.8)	1.220 (0.970–1.533)	0.089		
**Vascular invasion**					
No	254 (59.7)	Reference			
Yes	101 (65.1)	1.047 (0.831–1.318)	0.698		
**Multiple lobes**					
No	161 (55.5)	Reference		Reference	
Yes	194 (66.8)	1.474 (1.194–1.818)	< 0.001	1.180 (0.947–1.470)	0.14
**Primary tumor size**					
≤ 5 cm	58 (45.3)	Reference		Reference	
5–10 cm	129 (61.4)	1.527 (1.120–2.083)	0.008	1.369 (0.991–1.891)	0.057
≥ 10 cm	77 (69.3)	1.600 (1.137–2.250)	0.007	1.433 (1.006–2.042)	0.046
Unknown	91 (69.4)	2.412 (1.730–3.363)	< 0.001	1.618 (1.139–2.298)	0.007
**Chemotherapy**					
No	135 (57.6)	Reference		Reference	
Yes	220 (63.5)	0.754 (0.608–0.936)	< 0.011	0.607 (0.483–0.762)	< 0.001
**Radiation**					
No	304 (61.7)	Reference		Reference	
Yes	51 (57.9)	0.657 (0.488–0.884)	0.006	0.556 (0.409–0.756)	< 0.001
**Resection of the primary tumor**					
No	292 (68.0)	Reference		Reference	
Yes	63 (41.7)	0.277 (0.208–0.368)	< 0.001	0.305 (0.222–0.419)	< 0.001

### Propensity score matching

All characteristics between the surgery group and the non-surgery group were matched by PSM at a 1:1 ratio using the nearest neighbor method with a caliper of 0.01 based on the R package MatchIt. After propensity score matching, 100 patients were enrolled in both groups. The histograms of propensity scores showed that the two cohorts were well matched (Supplementary Fig. 1). Besides, after matching, no significant difference was revealed in all factors between the two groups (Supplementary Table S4).

**Figure 3:**
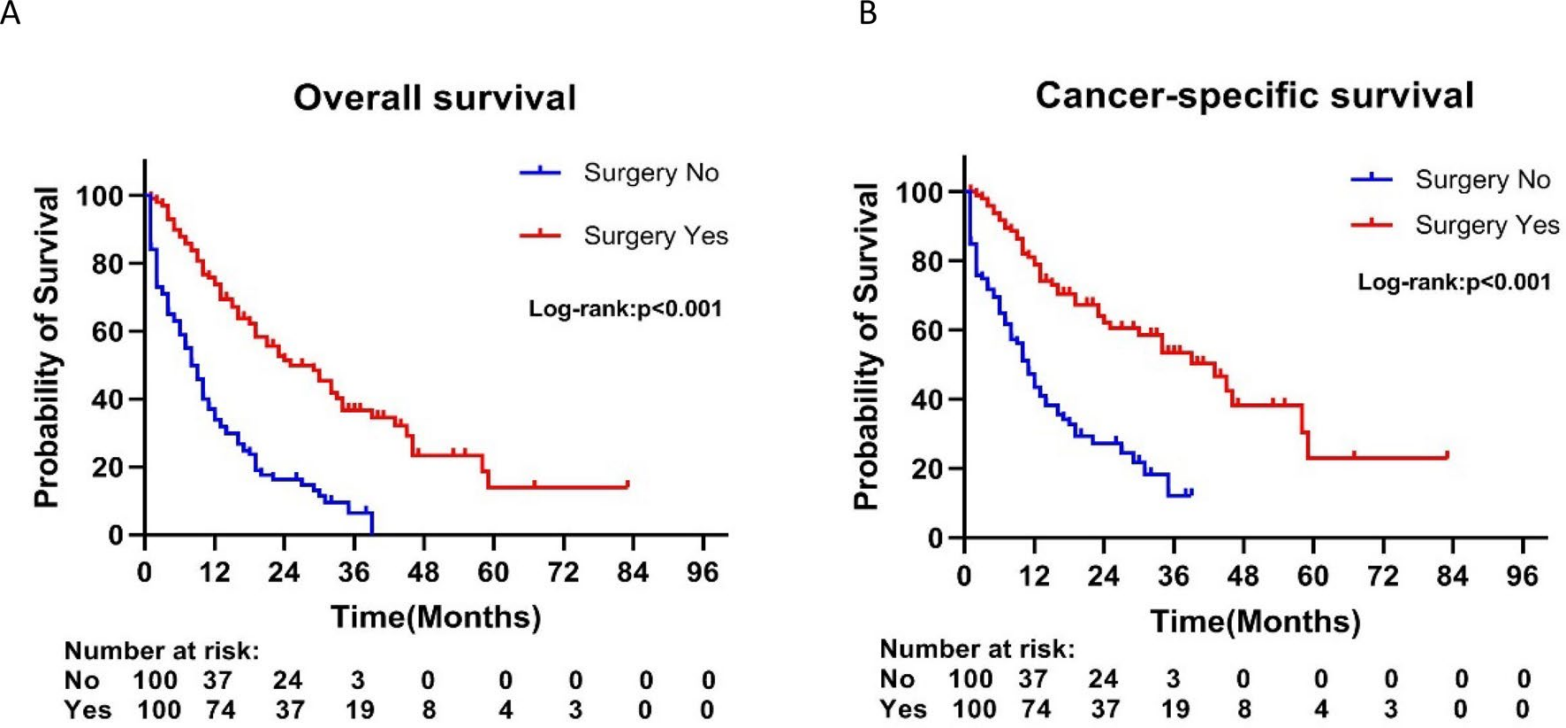
Kaplan–Meier curves of (**A**) overall and (**B**) cancer-specific survival according to whether patients underwent primary tumor surgery in the overall cohort after propensity score matching.

After PSM, the 5-year OS rate and median OS were 14.0% and 33 months in the surgery group, respectively, compared with 0% and 12 months in the non-surgery group (p < 0.001) (Fig. 3A). Meanwhile, the 5-year cancer-specific survival rate and median CSS of the surgery group were significantly better than those of the non-surgery group (5-year CSS rate: 22.9% vs. 0%, median CSS: 42 months vs. 15 months; p < 0.001) (Fig. 3B). Besides, in the PSM multivariate Cox regression model, primary tumor resection was still an independent prognostic factor to improve OS (HR = 0.315; 95% CI, 0.221–0.449; P < 0.001) and CSS (HR = 0.319; 95% CI: 0.210–0.486; P < 0.001) (Supplementary Table S5).

### Radiotherapy and chemotherapy in the surgery group

We found that compared with surgery alone, surgery combined with chemotherapy did not significantly improve the overall survival time of patients (median survival time:24 months vs. 32 months, p = 0.3769) (Supplementary Table S2). We reached a similar conclusion between the radiotherapy combined surgery group and the simple surgery group, with a median survival time of 30 months and 25 months, respectively (p = 0.4215). In the multivariate cox proportional hazards model, we found that the overall mortality rate of the chemotherapy group was comparable to the non-chemotherapy group in surgical patients (HR:1.353, 95% CI 0.813–2.253, p = 0.245), after adjusting for age at diagnosis, race, gender, marital status, insurance status, tumor size, tumor grade, lymph node status, vascular invasion, multiple liver lobes, and exposure to radiotherapy (Supplementary Table S6). Meanwhile, there was no significant difference in overall survival between the radiotherapy and non-radiotherapy groups after adjusting other variables using the multivariate cox proportional hazards model(HR:0.761, 95% CI 0.382–1.515, p = 0.436) (Supplementary Table S6).

**Figure 4:**
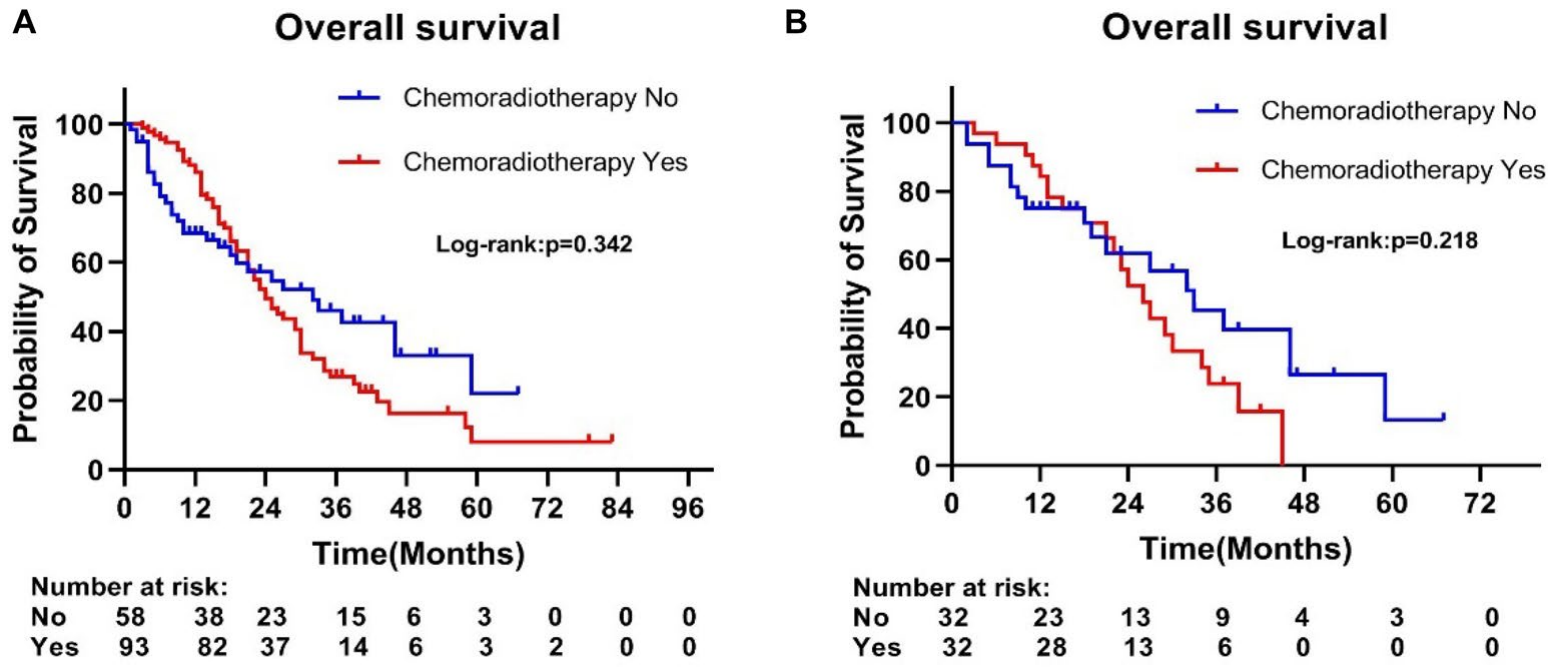
Kaplan–Meier curves of overall survival (**A**) before PSM and (**B**) after PSM according to whether patients underwent chemoradiotherapy in the surgery cohort.

Considering few radiotherapy patients in the operation group, we divided the operation group into chemoradiotherapy group and non-chemoradiotherapy group. Further, we analyzed the difference in OS between the two groups before and after PSM. Before matching, there were significant differences in age, gender, tumor size, lymph node status between the two groups (Supplementary Table S7). Meanwhile, there was no significant difference in OS between the chemoradiotherapy and non-chemoradiotherapy groups (median survival time: 33 months vs. 31 months, p = 0.342) (Fig. 4A). After matching, no significant difference was revealed in all factors between both groups (Supplementary Table S7); and the overall survival of the chemoradiotherapy group was similar to the non-chemoradiotherapy group (median survival time: 32 months vs. 29 months, p = 0.218) (Fig. 4B).

## Discussion

Multifocal ICCA, as well as extrahepatic metastasis, is often considered a contraindication for surgery, so palliative therapy is recommended. Because the surgical operation of multifocal ICCA means enlarging the volume of hepatectomy, which may lead to more postoperative liver failure and increase the risk of postoperative adverse events or perioperative death^15^. In addition, the existence of multiple lesions increases the possibility of R1 resection and recurrence rate, affecting the postoperative survival of patients^16^. Combined with the analysis of hepatectomy data over the past few decades, Mustafa et al. suggested that it was safe to resect large or multifocal tumors if sufficient liver residues were preserved^17^. Meanwhile, Gaya et al. found that compared with solitary ICCA, extended hepatectomy of multifocal ICCA was more frequent, but surgical resection of multiple lesions was still safe; there was no significant difference in the incidence of adverse events and perioperative mortality between the two groups^16^. Generally, in patients with normal liver function, the future liver remnant (FLR) > 20% is required, and in the case of impaired liver function, the more future liver remnant is required: the FLR of patients with fatty liver > 30%, the FLR of patients with liver cirrhosis > 40%^18^. Besides, in patients with insufficient residual liver volume, portal vein embolization (PVE) can make compensatory hyperplasia of the contralateral lobe in a short time and improve the possibility of R0 resection and the overall survival of the patients^19^. However, the effect of surgical resection on multifocal intrahepatic cholangiocarcinoma is still controversial. In order to further evaluate whether surgery will benefit this kind of ICCA with a poor prognosis, we conducted this retrospective analysis.

Previous studies showed that the postoperative survival rate of multifocal ICCA was lower than single lesion ICCA, with a 5-year OS of 8.6–18.7%^15–17^. However, these studies included only surgical patients and compared with single lesions, but not with unoperated multiple lesions. Our study found that primary tumor resection prolonged the median survival and improved the 1 -, 3- and 5-year survival rate in patients with multifocal ICCA, observed in both OS and CSS. Besides, multivariate Cox regression analysis further confirmed the effectiveness of surgery in treating multifocal intrahepatic cholangiocarcinoma; we found that surgical resection was an independent prognostic factor increasing OS and CSS. We analyzed the possible reasons why surgical resection can prolong the survival time of patients with multifocal ICCA. On the one hand, the possibility of continued invasion and progression of the primary lesion was removed after surgical resection. On the other hand, although the recurrence rate was as high as 60% after resection, it took a certain amount of time for new tumors to grow from scratch and affect patients' lives, which will also increase OS or CSS. From our results, surgical resection can prolong the survival time of patients with multiple lesions. However, we do not know the quality of life of these surviving patients, the tumor recurrence rate after resection, and the choice of specific treatment after recurrence. These may be important factors that affect our analysis of the results, so it is necessary to design a reasonable prospective study for further evaluation.

In the survival analysis of the surgical resection group, male, age > 65, tumor Grade III-IV and lymph node metastasis were important factors decreasing the OS; male, black race, tumor size > 10 cm, and lymph node metastasis had a worse CSS. It was found that men and African Americans have higher mortality rates and poor survival in the worldwide epidemiological survey of intrahepatic cholangiocarcinoma^20,21^. This was consistent with our results that males had poor OS and CSS and black people had poor CSS. However, the reasons for the poor survival prognosis of men and black people are not yet clear; it may be related to the unique characteristics (heredity, economic situation, etc.) of gender and race. Many studies have shown that lymph node metastasis is an important risk factor affecting the survival of intrahepatic cholangiocarcinoma^5,9,22,23^. Besides, related studies demonstrated that the number of lymph node metastasis was also related to survival and prognosis. Takahito et al. agreed that the survival rate of patients with positive lymph nodes > 3 was significantly lower than positive lymph nodes < 3^24^. Our study found that patients with tumor size > 10 cm had a poor prognosis, but the effect of tumor size on survival was controversial. In the eighth edition of the AJCC Cancer staging Manual, size has been listed as a prognostic factor for ICCA, and the only cut-off size considered is 5 cm in T1 tumors^20^. Some authors indicated that the cut-off value of 2 cm might identify very early tumors with low metastasis and recurrence rate^25^. Several authors' study has not found a significant correlation between tumor size and survival^5^. Omar et al. noted that the effect of tumor size on the risk of death was linear until the tumor size reached about 7 cm^26^. Besides, poorly differentiated tumors are more likely to metastasize and recur, leading to a worse prognosis. One study showed that poorly differentiated intrahepatic cholangiocarcinoma had a shorter survival time; the median OS of Grade I, Grade II, and Grade III were 73 months, 35 months, and 17 months, respectively (p = 0.001)^27^. Meanwhile, considering that older patients may be more likely to be complicated with other underlying diseases (such as cardio-cerebrovascular diseases), as well as insufficient physiological reserve capacity, the recovery ability is poor after surgical trauma, thus affecting their long-term prognosis. Therefore, surgical resection should be carried out cautiously after comprehensive evaluation in many aspects for these patients with high risk-factors.

The application of radiotherapy and chemotherapy in patients undergoing surgical resection can be divided into preoperative neoadjuvant therapy and postoperative adjuvant therapy. The efficacy of neoadjuvant therapy in patients with ICCA is controversial. Recent studies have shown that neoadjuvant therapy can reduce the risk of death and improve overall survival^28,29^, while some researchers believe that neoadjuvant is only associated with improved OS over upfront surgery in patients with resectable ICCA and high risk of treatment failure^30^. At present, most studies found that adjuvant therapy did not influence the prognosis of all ICCA patients following surgical resection; it was associated with a potential survival benefit in patients with high-risk features (such as positive margins, positive lymph nodes, or advanced T stage)^31–35^. However, the results of these studies are difficult to explain our findings, as they included all ICCA patients with surgical resection. Our study found that chemotherapy and radiotherapy did not significantly improve the survival prognosis of patients with multifocal intrahepatic cholangiocarcinoma in the surgical resection group, and the same conclusion was drawn in the subsequent multivariate cox regression analysis and survival analysis after PSM. However, our study did not identify whether patients received neoadjuvant therapy or adjuvant therapy, affecting our final results. At the same time, patients with surgical complications or postoperative deterioration of performance status may not be suitable for radiotherapy or chemotherapy. Regrettably, the basic information of postoperative complications and performance status have not been acquired, which may further affect our results. Therefore, for the application of radiotherapy and chemotherapy in multifocal ICCA patients with surgical resection, we need to carry out further prospective research to explore.

We must acknowledge some limitations of our research. First of all, our study is a retrospective study based on the SEER database, which may have led to selection bias. Surgeons tend to select patients with resectable tumors, better performance status, and good response to surgery. Although the multivariable Cox proportional hazards model and PSM have been used to reduce the selection bias, the hidden bias may still exist and lead to confounding. Second, the SEER database includes data from 19 states in the United States, but the data sources are limited to a single country, and it is worth further exploring whether our conclusions can be generalized to the whole world. Due to differences in economics and medical levels, intrahepatic cholangiocarcinoma in different regions may have different morbidity and mortality rates, and according to reports, Asians have the highest mortality rate^20^. Meanwhile, some important parameters such as the presence of cirrhosis, baseline tumor markers (CA-199), ECOG-PS (Eastern Cooperative Oncology Group performance status), liver function, surgical margins, recurrence and recurrence treatment, detailed radiotherapy and chemotherapy information, targeted therapy were not included because they were not available in the SEER registry, and which may have contributed to our findings. In addition, a recent study showed that tumor burden dictated the prognosis of resectable ICCA, and high tumor burden was closely related to poor OS^36^. Tumor burden was defined as the logarithm (natural) of tumor size plus the number of lesions [ICCA tumor burden = log_e_ (tumor size) + number of lesions]^36^. Regrettably, due to the lack of specific data on the number of tumors, it is impossible to calculate the tumor burden.

In conclusion, our study preliminarily demonstrates that surgery might have a beneficial effect in patients with multifocal intrahepatic cholangiocarcinoma. For these patients, resection of primary tumors is an appealing option, especially in the well-selected subgroups (female, age < 65, Grade I-II, negative lymph nodes, tumor size < 10 cm, non-black race). However, considering the limits of our study, more future prospective trials with large samples are needed to confirm these findings.

## Supplementary Information

Supplementary Information.

## Data Availability

Publicly available datasets were analyzed in this study. This data can be found here: Surveillance, Epidemiology, and End Results (SEER) database (https://seer.cancer.gov/).
