# Supplementary material for: Primary tumor resection improves survival in patients with multifocal intrahepatic cholangiocarcinoma based on a population study
Source: Sci Rep. 2021 Jun 9;11:12166. doi: 10.1038/s41598-021-91823-x (PMC8190174; doi:10.1038/s41598-021-91823-x)
Supplement: Supplementary file 1 — Supplementary Information. [file 41598_2021_91823_MOESM1_ESM.doc]

|  | AJCC v.7 | AJCC v.8 |
| --- | --- | --- |
| Primary tumor (T) |  |  |
| T1 | Solitary tumor without vascular invasion | / |
| T1a | / | Solitary tumor ≤5 cm without vascular invasion |
| T1b | / | Solitary tumor >5 cm without vascular invasion |
| T2 | / | Solitary tumor with intrahepatic vascular invasion or multiple tumors with or without vascular invasion |
| T2a | Solitary tumor with vascular invasion | / |
| T2b | Multiple tumors, with or without vascular invasion | / |
| T3 | Tumor perforating the visceral peritoneum or involving local hepatic structures by direct invasion | Tumor perforating the visceral peritoneum |
| T4 | Tumor with periductal invasion | Tumor involving local extrahepatic structures by direct invasion |
| Regional lymph nodes (N) |  |  |
| N0 | No regional lymph node metastasis | No regional lymph node metastasis |
| N1 | Regional lymph node metastasis present | Regional lymph node metastasis present |
| Distant metastasis (M) |  |  |
| M0 | No distant metastasis | No distant metastasis |
| M1 | Distant metastasis present | Distant metastasis present |
| TNM stage |  |  |
| I | T1, N0, M0 | / |
| Ia | / | T1a, N0, M0 |
| Ib | / | T1b, N0, M0 |
| II | T2a/b, N0, M0 | T2, N0, M0 |
| III | T3, N0, M0 | / |
| IIIa | / | T3, N0, M0 |
| IIIb | / | T4, Any N, M0 or Any T, N1, M0 |
| IV | / | Any T, any N, M1 |
| IVa | T4, N0, M0 or Any T, N1, M0 | / |
| IVb | Any T, any N, M1 | / |

**Supplementary Table S1**: Current staging of cholangiocarcinoma (AJCC 7th and 8th Edition)

| Variables | Median survival, months | HR (95% CI) | P value（Log-rank test） |
| --- | --- | --- | --- |
| Age group, years |  |  |  |
| <65 | 30 | Reference | 0.0195 |
| ≥65 | 22 | 1.588(1.048-2.405) |
| Race |  |  |  |
| White | 27 | Reference | 0.185 |
| Black | 17 | 1.875(0.762-4.611) |
| Others | 22 | 0.930(0.523-1.657) |
| Gender |  |  |  |
| Male | 19 | Reference | 0.0027 |
| Female | 30 | 0.523(0.342-0.799) |
| Insurance status |  |  |  |
| Uninsured | 18 | Reference | 0.219 |
| Insured | 25 | 0.749(0.313-1.794) |
| Marital status |  |  |  |
| Unmarried | 21 | Reference | 0.588 |
| Married | 25 | 0.887(0.567-1.390) |
| Grade |  |  |  |
| Grade I -II | 30 | Reference | 0.029 |
| Grade III-IV | 22 | 1.635(0.967-2.764) |
| LN metastases |  |  |  |
| No | 30 | Reference | 0.0068 |
| Yes | 18 | 1.836(1.065-3.167) |
| Vascular invasion |  |  |  |
| No | 27 | Reference | 0.358 |
| Yes | 24 | 1.225(0.795-1.887) |
| Multiple lobes |  |  |  |
| No | 25 | Reference | 0.367 |
| Yes | 29 | 0.8124(0.515-1.280) |
| Primary tumor size |  |  |  |
| ≤5 cm | 27 | Reference | 0.6328 |
| 5-10 cm | 27 | 1.060(0.651-1.727) |
| ≥10 cm | 24 | 1.344(0.752-2.403) |
| Chemotherapy |  |  |  |
| No | 32 | Reference | 0.3769 |
| Yes | 24 | 1.205(0.795-1.825) |
| Radiation |  |  |  |
| No | 25 | Reference | 0.4215 |
| Yes | 30 | 0.784(0.449-1.368) |

**Supplementary Table S2**：Subgroup analysis of OS in surgical group

| Variables | Median survival, months | HR (95% CI) | P value（Log-rank test） |
| --- | --- | --- | --- |
| Age group, years |  |  |  |
| <65 | 40 | Reference | 0.6904 |
| ≥65 | 39 | 1.104(0.665-1.832) |
| Race |  |  |  |
| White | 39 | Reference |  |
| Black | 24 | 2.308(0.755-7.056) | 0.0316 |
| Others | 40 | 1.255(0.618-2.551) | 0.4927 |
| Gender |  |  |  |
| Male | 29 | Reference | 0.0138 |
| Female | 46 | 0.545(0.329-0.904） |
| Insurance status |  |  |  |
| Uninsured | 39 | Reference | 0.8182 |
| Insured | 46 | 0.889(0.306-2.584) |
| Marital status |  |  |  |
| Unmarried | 34 | Reference | 0.7527 |
| Married | 40 | 0.918(0.532-1.583) |
| Grade |  |  |  |
| Grade I -II | 43 | Reference | 0.0614 |
| Grade III-IV | 25 | 1.735(0.921-3.270) |
| LN metastases |  |  |  |
| No | 45 | Reference | 0.0065 |
| Yes | 21 | 2.050(1.069-3.933) |
| Vascular invasion |  |  |  |
| No | 43 | Reference | 0.1216 |
| Yes | 30 | 1.472(0.878-2.468) |
| Multiple lobes |  |  |  |
| No | 34 | Reference | 0.3326 |
| Yes | 29 | 1.336(0.769-2.320) |
| Primary tumor size |  |  |  |
| ≤5 cm | 42 | Reference |  |
| 5-10 cm | 40 | 1.166(0.632-2.150) | 0.6211 |
| ≥10 cm | 24 | 2.072(1.051-4.085) | 0.0199 |
| Chemotherapy |  |  |  |
| No | 30 | Reference | 0.0735 |
| Yes | 39 | 0.615(0.371-1.020) |
| Radiation |  |  |  |
| No | 37 | Reference | 0.6986 |
| Yes | 39 | 0.872(0.445-1.708) |

**Supplementary Table S3**：Subgroup analysis of CSS in surgical group

| Variables | No primary tumor resection n =100 | Primary tumor resection  n =100 | p-value |
| --- | --- | --- | --- |
| Age group, years, % |  |  |  |
| <65 | 47(47%) | 51(51%) | 0.572 |
| ≥65 | 53(53%) | 49(49%) |
| Race, % |  |  |  |
| White | 75(75%) | 80(80%) | 0.698 |
| Black | 9(9%) | 7(7%) |
| Others | 16(16%) | 13(13%) |
| Gender, % |  |  |  |
| Male | 42(42%) | 48(48%) | 0.394 |
| Female | 58(58%) | 52(52%) |
| Insurance status, % |  |  |  |
| Uninsured | 7(7%) | 8(8%) | 0.607 |
| Insured | 92(92%) | 92(92%) |
| Unknown | 1(1%) | 0(0%) |
| Marital status, % |  |  |  |
| Unmarried | 29(29%) | 34(34%) | 0.279 |
| Married | 66(66%) | 64(64%) |
| Unknown | 5(5%) | 2(2%) |
| Grade, % |  |  |  |
| Grade I -II | 34(34%) | 40(40%) | 0.452 |
| Grade III-IV | 30(30%) | 27(27%) |
| Unknown | 36(36%) | 33(33%) |
| LN metastases, % |  |  |  |
| No | 74(74%) | 76(76%) | 0.744 |
| Yes | 26(26%) | 24(24%) |
| Chemotherapy, % |  |  |  |
| No | 46(46%) | 40(40%) | 0.392 |
| Yes | 54(54%) | 60(60%) |
| Radiation, % |  |  |  |
| No | 82(82%) | 84(84%) | 0.707 |
| Yes | 18(18%) | 16(16%） |
| Vascular invasion, % |  |  |  |
| No | 67(67%) | 64(64%) | 0.655 |
| Yes | 33(33%) | 36(36%) |
| Multiple lobes, % |  |  |  |
| No | 74(74%) | 67(67%) | 0.279 |
| Yes | 26(26%) | 33(33%) |
| Primary tumor size, % |  |  |  |
| ≤5 cm | 31(31%) | 30(30%) | 0.939 |
| 5-10 cm | 42(42%) | 40(40%) |
| ≥10 cm | 17(17%) | 23(23%) |
| Unknown | 10(10%) | 7(7%) |

**Supplementary Table S4:** Baseline characteristics of patients with multifocal intrahepatic cholangiocarcinoma after PSM

| Characteristic | Overall survival | | | | Cancer-specific survival | | | |
| --- | --- | --- | --- | --- | --- | --- | --- | --- |
|  | Univariable | | Multivariable | | Univariable | | Multivariable | |
|  | HR (95% CI) | p-value | HR (95% CI) | p-value | HR (95% CI) | p-value | HR (95% CI) | p-value |
| Age group, years |  |  |  |  |  |  |  |  |
| <65 | Reference |  |  |  | Reference |  |  |  |
| ≥65 | 1.262(0.911-1.750) | 0.162 |  |  | 1.040(0.710-1.524) | 0.84 |  |  |
| Race |  |  |  |  |  |  |  |  |
| White | Reference |  |  |  | Reference |  |  |  |
| Black | 1.723(0.985-3.011) | 0.056 |  |  | 2.281(1.262-4.120) | 0.006 | 3.373(1.794-6.344) | < 0.001 |
| Others | 1.032(0.653-1.632) | 0.892 |  |  | 1.315(0.794-2.178) | 0.287 | 1.396(0.833-2.338) | 0.205 |
| Gender |  |  |  |  |  |  |  |  |
| Female | Reference |  | Reference |  | Reference |  |  |  |
| Male | 1.385(1.002-1.914) | 0.049 | 1.421(1.021-1.978) | 0.037 | 1.321(0.905-1.926) | 0.149 |  |  |
| Insurance status |  |  |  |  |  |  |  |  |
| Uninsured | Reference |  |  |  | Reference |  |  |  |
| Insured | 0.715(0.403-1.268) | 0.251 |  |  | 0.865(0.420-1.784) | 0.695 |  |  |
| Unknown | / | / |  |  | / | / |  |  |
| Marital status |  |  |  |  |  |  |  |  |
| Unmarried | Reference |  |  |  | Reference |  |  |  |
| Married | 1.100(0.773-1.565) | 0.595 |  |  | 1.104(0.733-1.661) | 0.637 |  |  |
| Unknown | 0.984(0.417-2.320) | 0.971 |  |  | 0.668(0.204-2.192) | 0.506 |  |  |
| Grade |  |  |  |  |  |  |  |  |
| Grade I -II | Reference |  | Reference |  | Reference |  | Reference |  |
| Grade III-IV | 1.431(0.942-2.175) | 0.093 | 1.510(0.989-2.306) | 0.056 | 1.938(1.165-3.225) | 0.011 | 2.119(1.258-3.569) | 0.005 |
| Unknown | 1.621(1.103-2.383) | 0.014 | 1.598(1.082-2.360) | 0.018 | 2.285(1.429-3.652) | 0.001 | 2.126(1.307-3.458) | 0.002 |
| LN metastases |  |  |  |  |  |  |  |  |
| No | Reference |  |  |  | Reference |  | Reference |  |
| Yes | 1.302(0.900-1.883) | 0.161 |  |  | 1.532(1.015-2.313) | 0.042 | 1.726(1.105-2.696) | 0.016 |
| Vascular invasion |  |  |  |  |  |  |  |  |
| No | Reference |  |  |  | Reference |  | Reference |  |
| Yes | 1.372(0.981-1.220) | 0.985 |  |  | 1.675(1.143-2.454) | 0.008 | 2.103(1.393-3.177) | < 0.001 |
| Multiple lobes |  |  |  |  |  |  |  |  |
| No | Reference |  |  |  | Reference |  |  |  |
| Yes | 1.359(1.135-1.918) | 0.064 |  |  | 0.891(0.589-1.346) | 0.583 |  |  |
| Primary tumor size |  |  |  |  |  |  |  |  |
| ≤5 cm | Reference |  |  |  | Reference |  |  |  |
| 5-10 cm | 0.974(0.657-1.443) | 0.895 |  |  | 1.356(0.832-2.210) | 0.222 |  |  |
| ≥10 cm | 0.881(0.558-1.391) | 0.587 |  |  | 1.316(0.763-2.271) | 0.324 |  |  |
| Unknown | 1.260(0.688-2.305) | 0.454 |  |  | 1.718(0.843-3.504) | 0.136 |  |  |
| Chemotherapy |  |  |  |  |  |  |  |  |
| No | Reference |  | Reference |  | Reference |  | Reference |  |
| Yes | 0.705(0.508-0.979) | 0.037 | 0.650(0.463-0.912) | 0.013 | 0.831(0.565-1.224) | 0.349 | 0.557(0.366-0.846) | 0.006 |
| Radiation |  |  |  |  |  |  |  |  |
| No | Reference |  |  |  | Reference |  |  |  |
| Yes | 0.846(0.554-1.293) | 0.441 |  |  | 0.679(0.399-1.156) | 0.154 |  |  |
| Resection of the primary tumor |  |  |  |  |  |  |  |  |
| No | Reference |  | Reference |  | Reference |  | Reference |  |
| Yes | 0.329(0.232-0.467) | < 0.001 | 0.315(0.221-0.449) | < 0.001 | 0.311(0.206-0.470) | < 0.001 | 0.319(0.210-0.486) | < 0.001 |

**Supplementary Table S5:** Factors associated with overall and cancer-specific survival of patients with multifocal intrahepatic cholangiocarcinoma after PSM

| Characteristic | Univariable | |
| --- | --- | --- |
|  | HR (95% CI) | p-value |
| Age group, years |  |  |
| <65 | Reference |  |
| ≥65 | 1.909(1.205-3.025) | 0.006 |
| Race |  |  |
| White | Reference |  |
| Black | 3.399(1.599-7.225) | 0.001 |
| Others | 1.083(0.587-1.997) | 0.799 |
| Gender |  |  |
| Female | Reference |  |
| Male | 1.949(1.241-3.060） | 0.004 |
| Insurance status |  |  |
| Uninsured | Reference |  |
| Insured | 0.369(0.160-0.849) | 0.019 |
| Unknown | 0.502(0.056-4.518) | 0.539 |
| Marital status |  |  |
| Unmarried | Reference |  |
| Married | 0.697(0.423-1.147) | 0.155 |
| Unknown | 0.623(0.171-2.279) | 0.475 |
| Grade |  |  |
| Grade I -II | Reference |  |
| Grade III-IV | 1.591(0.953-2.654) | 0.076 |
| Unknown | 2.606(1.535-4.423) | < 0.001 |
| LN metastases |  |  |
| No | Reference |  |
| Yes | 2.875(1.699-4.867) | < 0.001 |
| Vascular invasion |  |  |
| No | Reference |  |
| Yes | 1.256(0.788-2.002) | 0.339 |
| Multiple lobes |  |  |
| No | Reference |  |
| Yes | 0.622(0.365-1.061) | 0.081 |
| Primary tumor size |  |  |
| ≤5 cm | Reference |  |
| 5-10 cm | 1.079(0.622-1.936) | 0.749 |
| ≥10 cm | 1.096(0.582-2.067) | 0.776 |
| Unknown | 1.464(0.527-4.062) | 0.464 |
| Chemotherapy |  |  |
| No | Reference |  |
| Yes | 1.353(0.813-2.253) | 0.245 |
| Radiation |  |  |
| No | Reference |  |
| Yes | 0.761(0.382-1.515) | 0.436 |

**Supplementary Table S6:** Multivariate cox proportional hazards model in surgical patients with multifocal intrahepatic cholangiocarcinoma

|  | Before PSM | |  | After PSM | |  |
| --- | --- | --- | --- | --- | --- | --- |
| Variables | Chemoradiotherapy n =93 | non-chemoradiotherapy n =58 | p-value | Chemoradiotherapy n =32 | non-chemoradiotherapy n =32 | p-value |
| Age group, years, % |  |  |  |  |  |  |
| <65 | 61(65.6%) | 17(29.3%) | <0.001 | 12(37.5%) | 13(40.6%) | 0.798 |
| ≥65 | 32(34.4%) | 41(70.7%) |  | 20(62.5%) | 19(59.4%) |  |
| Race, % |  |  |  |  |  |  |
| White | 74(79.6%) | 43(74.1%) | 0.421 | 26(81.3%) | 27(84.4%) | 0.546 |
| Black | 7(7.5%) | 5(8.6%) |  | 2(6.3%) | 3(9.4%) |  |
| Others | 12(12.9%) | 10(17.2%) |  | 4(12.5%) | 2(6.3%) |  |
| Gender, % |  |  |  |  |  |  |
| Male | 36(38.7%) | 33(56.9%) | 0.03 | 16(50.0%) | 15(46.9%) | 0.803 |
| Female | 57(61.3%) | 25(43.1%) |  | 16(50.0%) | 17(53.1%) |  |
| Insurance status, % |  |  |  |  |  |  |
| Uninsured | 3(3.2%) | 6(10.3%) | 0.066 | 3(9.4%) | 4(12.5%) | 0.69 |
| Insured | 89(95.7%) | 52(89.7%) |  | 29(90.6%) | 28(87.5%) |  |
| Unknown | 1(1.1%) | 0(0%) |  | 0(0%) | 0(0%) |  |
| Marital status, % |  |  |  |  |  |  |
| Unmarried | 27(29.0%) | 18(31.0%) | 0.516 | 8(25.0%) | 9(28.1%) | 0.799 |
| Married | 61(65.6%) | 39(67.2%) |  | 23(71.9%) | 22(68.8%) |  |
| Unknown | 5(5.4%) | 1(1.7%) |  | 1(3.1%) | 1(3.1%) |  |
| Grade, % |  |  |  |  |  |  |
| Grade I -II | 43(46.2%) | 28(48.3%) | 0.563 | 16(50.0%) | 18(56.3%) | 0.875 |
| Grade III-IV | 31(33.3%) | 15(25.9%) |  | 11(34.4%) | 6(18.8%) |  |
| Unknown | 19(20.4%) | 15(25.9%) |  | 5(15.6%) | 8(25.0%) |  |
| LN metastases, % |  |  |  |  |  |  |
| No | 63(67.7%) | 54(93.1%) | 0.001 | 27(84.4%) | 28(87.5%) | 0.72 |
| Yes | 30(32.3%) | 4(6.9%) |  | 5(15.6%) | 4(12.5%) |  |
| Vascular invasion, % |  |  |  |  |  |  |
| No | 55(59.1%) | 40(69%) | 0.225 | 18(56.3%) | 19(59.4%) | 0.801 |
| Yes | 38(40.9%) | 18(31%) |  | 14(43.8%) | 18(40.6%) |  |
| Multiple lobes, % |  |  |  |  |  |  |
| No | 66(71.0%) | 67(67%) | 0.279 | 23(71.9%) | 23(71.9%) | 1 |
| Yes | 27(29.0%) | 33(33%) |  | 9(28.1%) | 9(28.1%) |  |
| Primary tumor size, % |  |  |  |  |  |  |
| ≤5 cm | 23(24.7%) | 35(60.3%) | 0.001 | 12(37.5%) | 14(43.8%) | 0.878 |
| 5-10 cm | 44(47.3%) | 15(25.9%) |  | 16(50.0%) | 12(37.5%) |  |
| ≥10 cm | 21(22.6%) | 6(10.3%) |  | 3(9.4%) | 4(12.5%) |  |
| Unknown | 5(5.4%) | 2(3.4%) |  | 5(3.1%) | 2(6.3%) |  |

**Supplementary Table S7:** Characteristics of patients with multifocal intrahepatic cholangiocarcinoma in surgery group


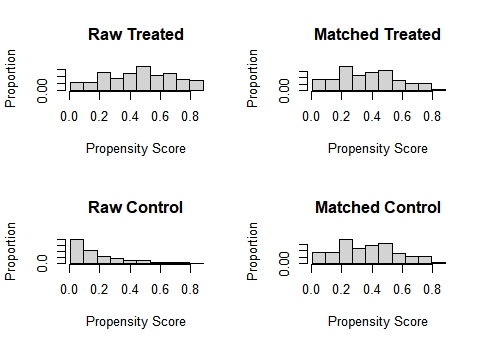


**Supplementary** **Figure 1**: The histograms of propensity scores for the raw and matched case and controls.
